# Chest wall loading in the ICU: pushes, weights, and positions

**DOI:** 10.1186/s13613-022-01076-8

**Published:** 2022-11-08

**Authors:** John Selickman, John J. Marini

**Affiliations:** 1 Department of Pulmonary and Critical Care Medicine, University of Minnesota, Minneapolis, MN USA; 2 Department of Critical Care Medicine, Regions Hospital, MS 11203B, 640 Jackson St., St. Paul, MN 55101-2595 USA

**Keywords:** ARDS, Ventilator-induced lung injury, Mechanical ventilation, Respiratory mechanics, Chest wall

## Abstract

Clinicians monitor mechanical ventilatory support using airway pressures—primarily the plateau and driving pressure, which are considered by many to determine the safety of the applied tidal volume. These airway pressures are influenced not only by the ventilator prescription, but also by the mechanical properties of the respiratory system, which consists of the series-coupled lung and chest wall. Actively limiting chest wall expansion through external compression of the rib cage or abdomen is seldom performed in the ICU. Recent literature describing the respiratory mechanics of patients with late-stage, unresolving, ARDS, however, has raised awareness of the potential diagnostic (and perhaps therapeutic) value of this unfamiliar and somewhat counterintuitive practice. In these patients, interventions that reduce resting lung volume, such as loading the chest wall through application of external weights or manual pressure, or placing the torso in a more horizontal position, have unexpectedly improved tidal compliance of the lung and integrated respiratory system by reducing previously undetected end-tidal hyperinflation. In this interpretive review, we first describe underappreciated lung and chest wall interactions that are clinically relevant to both normal individuals and to the acutely ill who receive ventilatory support. We then apply these physiologic principles, in addition to published clinical observation, to illustrate the utility of chest wall modification for the purposes of detecting end-tidal hyperinflation in everyday practice.

## Introduction

Clinicians usually monitor mechanical ventilatory support using airway pressures—primarily the plateau and driving pressure, recorded during passive inflation. Indeed, the focus of lung-protective ventilation centers on the numerical values of these airway pressures, which are considered by many to determine the risk or safety of the applied tidal volume (*V*_T_) [1]. Although the series-coupled lung and chest wall share a common volume, and, therefore, jointly determine not only the transpulmonary pressure (*P*_L_) that distends the lung but also the airway pressures used to guide ventilation, the important influence of the chest wall is often discounted or ignored altogether outside such clinical extremes as morbid obesity, severe skeletal deformity, or abdominal compartment syndrome.

Actively limiting chest wall expansion through chest wall loading (applying weights or pressures to its surface) simultaneously *restricts* lung expansion. Doing so for diagnostic or therapeutic purposes is seldom performed in the clinical setting, with several exceptions. Scattered reports, for example, indicate that external compression of the chest wall to alleviate hyperinflation may be a temporizing and life-saving measure for status asthmaticus [2, 3]. While the horizontal prone position, a form of chest wall loading to the ventral body surface, is used in acute respiratory distress syndrome (ARDS) to even the distribution of transpulmonary pressures, few advocate external chest wall compression for the explicit purpose of restricting lung expansion in that setting. Quite the opposite, applying high levels of positive end-expiratory pressure (PEEP) and semi-upright positioning are generally considered beneficial when seeking to enlarge the aerated lung volume by recruiting additional lung units [4–6]. This interpretation generally holds merit for the massively obese and during the initial phase of ARDS, especially for those with overtly edematous, recruitable lungs.

Yet, recent literature describing the mechanics of severe ARDS, primarily in patients with late-stage ARDS secondary to COVID-19, has raised awareness of the potential diagnostic (and perhaps therapeutic) value of doing the polar opposite, i.e., *reducing* PEEP, positioning the torso more horizontally, and loading the chest wall through application of external weights or manual pressures to the body surface [7–14]. Even though such measures invariably reduce the resting lung volume, they may improve the tidal compliance of the lung and integrated respiratory system when undetected end-tidal hyperinflation is extensive and recruitable lung tissue is relatively less [15]. We refer to this phenomenon, whereby compression—or loading, of the chest wall in the presence of undetected end-tidal hyperinflation results in *improved* compliance of lungs and integrated respiratory system (and vice versa, whereby unloading of the chest wall results in *worsened* compliance of the lungs and integrated respiratory system), as mechanical ‘paradox’. Detecting its presence may hold potential for therapeutic interventions.

Our purpose in this interpretive review is twofold. We first describe underappreciated lung and chest wall interactions that are clinically relevant to both normal individuals and the acutely ill who receive ventilatory support. We then apply these physiologic principles in addition to published clinical observations to illustrate the utility of chest wall modification for the purposes of detecting end-tidal hyperinflation in everyday practice.

## Essential physiology relevant to chest wall modification

Anatomically, the chest wall is formed by two interacting compartments above and below the diaphragm that jointly influence pressures within the pleural cavity. The forces of inflation can be viewed as expanding the ribs while simultaneously depressing the diaphragm against the opposing pressure of the abdomen. Under passive conditions, these compartments unavoidably interact with one another, distending or contracting in relative proportion to their individual compliances, which differ with pathology, body habitus, age, lung volume, body position, and gravitational gradients. Because externally imposed loads on the supine chest wall are applied selectively to only one of these diaphragm-separated compartments (either the rib cage or abdomen), it is helpful to consider the characteristics of each, and how the distribution of compartmental compliances varies with weights, manual pressure and body positioning.

### Compartmental compliance

In healthy normal subjects, the inflation compliance of the rib cage (*C*_RC_) is several times greater than that of the abdomen (*C*_AB_) [16]; together, they determine the integrated compliance of the chest wall (*C*_CW_). Unlike the gas-filled supradiaphragmatic compartment, the volume of the abdomen itself is essentially fixed but may change its shape, diaphragm-bounded position, and flexibility. Chest wall compliance deteriorates with advancing age, due primarily (but not exclusively) to stiffening of the rib cage [16]. Interestingly, *C*_CW_ changes relatively little for face forward (supine) postures over the range of 0° to 90°, even as the resting lung volume (functional residual capacity, FRC) and partitioned components (*C*_RC_ and *C*_AB_) of *C*_CW_ change markedly [17]. The positional loss of FRC and expiratory reserve during reclining comes exclusively at the expense of the rib cage compartment, whose functional compliance falls significantly when horizontal, while that of the abdomen increases [18].

**Fig. 1:**
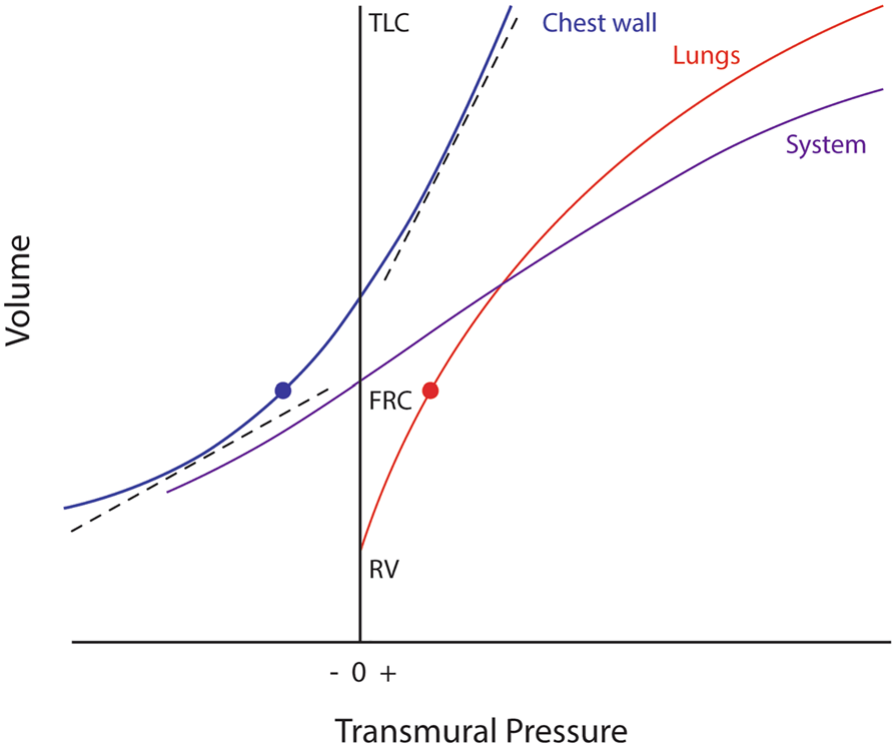
Pressure–volume relationships of the respiratory system. The inflation compliance of the chest wall is high throughout the range extending from functional residual capacity (FRC) to total lung capacity (TLC) (upper dashed line) but flattens as it approaches residual volume (RV) (lower dashed line)

The inflation compliance of the normal chest wall remains relatively high and changes little throughout much of the volume range that extends from FRC to total lung capacity [18]. However, at very low lung and very high thoracic volumes approaching residual volume and total lung capacity, respectively, the pressure–volume relationship of the chest wall flattens [19] (Fig. 1). For example, when compressed below its normal resting position without PEEP, the rib cage springs outward and stiffens remarkably [16]; *C*_RC_ (and by extension compliance of the respiratory system, *C*_RS_) falls progressively in response increasing compression, whereas *C*_AB_ does not until the diaphragm is pushed to its tethered limit. Neither extreme of low chest wall compliance is entered under usual clinical conditions when managing ARDS using lung protective strategies. Notably, the *aerated* volume of the lung in ARDS (the ‘baby lung’) is much lower than that occupied by the infiltrated, edematous entire lung, which tends to remain normally distended at FRC, as does the chest wall. It follows, therefore, that the aerated baby lung and chest wall operate from different points on their respective pressure–volume curves. The inherent compliance of the chest wall in ARDS also depends on the causative disease process, tending to be lower in extrapulmonary lung injury than in pneumonia [20].

By contrast with ARDS, the hyperinflated airspaces of exacerbated asthma and COPD are surrounded by an equally distended chest wall. Estimates made in such patients suggest low inflation compliance of a quasi-normal chest wall that often encroaches on its upper inflection zone (low *C*_CW_) during tidal breathing [21].

### Influence of body position on lung volume

Gravitational forces exert an important influence on lung volumes, ventilation distribution, and *C*_RS_. Under most circumstances, more upright positioning substantially increases both *C*_CW_ and FRC. In normal subjects, reclining decreases FRC, primarily due to the upward pressure of the abdominal contents on the diaphragm and compression of some dorsal lung segments by the combined weight of the heart and mediastinum [22]. FRC declines by approximately 30% (600–900 mL) when shifting from the sitting to the horizontal supine position [23]. If one assumes normal supine *C*_RS_ (approximately 80 mL/cmH_2_O), 5 to 8 cmH_2_O PEEP may be needed simply to offset volume losses of upright FRC associated with this positional change.

Patients with severe airflow obstruction generally lose much less total volume than do normal subjects of similar age when assuming supine recumbency, in part due to air-trapping that occurs as a result of extensive airway closure through part or all of the breathing cycle [23]; in such patients, a reduced number of lung units remain open throughout expiration in the horizontal position, resulting in a smaller functioning lung with diminished capacity to eliminate CO_2_ efficiently [23].

In massive obesity (body mass index > 40 kg/m^2^), the expiratory reserve volume (ERV, the FRC minus the residual volume) may nearly disappear even when fully upright due to loading of the chest wall by adipose tissue [24]. Depleted of the ERV, changes in end-expiratory aerated lung volume during the transition from sitting to recumbent may actually be minimal. In addition, reversible small airway collapse and gas-trapping occur as *P*_L_ falls below a critical threshold during the tidal deflation phase [25–27]. Tidal gas trapping at the small airway level (independent of alveolar collapse) has also been described in supine patients with ARDS, especially in those ventilated with low PEEP [28, 29].

### Positional redistribution of lung volume

Recumbency redistributes lung volume by altering the geometry of the thoracic shell and its contents. When supine and horizontal, the heart and mediastinal contents more directly compress the left than the right lower lobe bronchi. This asymmetric anatomy helps explain the tendency for atelectasis to develop more commonly in the left lower lobe in post-operative and bedridden patients—especially in those with cardiomegaly [30]. Because the pleural pressures in gravitationally dependent zones exceed those in non-dependent zones, local *P*_L_ and alveolar volumes are lower in those regions [31]. Consequently, for semi-recumbent supine patients with heavy, edematous lungs (e.g., ARDS) an intensified gravitational gradient of pleural pressure (*P*_pl_) accentuates the tendency for dorsal and peri-diaphragmatic atelectasis and consolidation [31, 32].

The gradient of *P*_pl_ is lower when prone than supine, largely due to positional reshaping of the thoracic cavity and offloading the weight of the heart and mediastinal contents [33]. The supporting surface compresses the anterior chest and abdomen when prone, causing overall *C*_CW_ to decline. Although conversion from the supine to prone position in ARDS is usually accompanied by marginal net changes of total resting lung volume (< 15%), the distribution of compressive regional atelectasis changes significantly. As the larger, well-perfused, and previously compressed dorsal regions become better aerated, oxygenation in ARDS usually improves [33]. While less extensively studied in non-ARDS conditions, benefits from prone positioning with respect to gas exchange and mechanics have also been reported in cardiogenic pulmonary edema [34] and severe airflow obstruction [35].

### Intercompartmental pressure transmission

Transmission of extrapulmonary pressure increments between the rib cage and abdomen that occur naturally or during chest wall loading is a complex function of body position, lung volume, and the relative distensibility of these compartments. Selective compartmental pressure increases are reflected to a varying extent on the other side of the diaphragmatic barrier [36]. For example, during passive tidal breathing, *P*_pl_ increases minimally as intra-abdominal pressures (IAPs) rise over a range from 0 to 10 cmH_2_O. However, experimental studies conducted in healthy anesthetized pigs indicate that once IAP rise above its upper baseline value of ≈ 10 cmH_2_O, approximately half of any further incremental increase of IAP transmits to the pleural space at the end of inflation, raising the plateau and driving pressures associated with a fixed *V*_T_ [37]. Transmission of IAP in healthy human subjects appears to be similar [38].

As IAP rises, contraction of the resting lung volume occurs via the open circuit during the deflation phase of tidal breathing, preventing major increases of end-expiratory *P*_pl_, even at rather high levels of IAP [37]. Factors that increase the IAP transmission fraction during inflation are higher lung volumes, more horizontal positioning, and increased rib cage stiffness. At very high IAP, further increases in *P*_pl_ are limited by a domed diaphragm stretched to its rib-tethered limits [39].

*P*_pl_ increases that originate within the supra-diaphragmatic compartment arise naturally from expansion of the lungs or from large pleural effusions. These generally exert only modest effects on IAP. The rising *P*_pl_ that develops during passive inflation, for example, causes IAP to elevate by a smaller amount that varies inversely with the ratio of *C*_RC_ to *C*_AB_.

### Hemodynamics

The effect of chest wall loading on hemodynamics depends on the site—above or below the diaphragm—at which loading occurs. Selective increases of *P*_pl_ that originate from loads above the diaphragm exert two actions that may influence cardiac performance:

1. Venous return depends on the pressure gradient between the ‘upstream’ mean systemic pressure, influenced heavily by the venous reservoir below the diaphragm, and the central venous (or ‘downstream’) pressure located within the thoracic cavity. Increased *P*_pl_ raises right atrial pressure and tends to decrease venous return as higher intraluminal pressures upstream simultaneously fill the extensive capacitance vessels of the splanchnic bed and lower extremities [40, 41]. Unless the mean systemic to central venous pressure difference is maintained by increased vascular tone or administration of adequate intravascular fluid, cardiac output may fall [42]. Lacking adequate compensation mechanisms, transition to an upright position may also favor a drop in venous return, due to pooling of blood in the aforementioned capacitance vessels [43].
2. Selective increases of *P*_pl_ above the diaphragm also reduce left ventricular afterload, although assuming cardiac contractility is not markedly impaired, this potential benefit is of low magnitude and relatively inconsequential.

In contrast, the effect of selectively increasing IAP is variable. Modest increases in IAP tend to increase venous return due to a transfer of blood volume from splanchnic capacitance vessels to the central veins [44], a shift which may hinder therapeutic objectives by excessively preloading the right ventricle or augmenting lung edema across the permeable vessels of ARDS [45]. As IAP rises further, compression of the inferior vena cava may raise venous resistance, decreasing both venous return and cardiac output [44]. Elevations in IAP also displace the relaxed diaphragm cephalad, promoting basilar atelectasis and impairing oxygenation that, unless offset by adequate PEEP, will increase right ventricular afterload.

## Methods for chest wall modification in the ICU

In non-acute care settings, binding of the abdomen or chest wall may help improve the efficiency of breathing efforts in patients with certain neuromuscular diseases, for example, diaphragmatic paralysis [46, 47]. Binding of the chest wall is also employed in surgical trauma to help stabilize flail segments, sucking chest wounds, or movement-induced pain. In the medical ICU, however, attempts to modify the lungs’ enclosure are generally limited to changes of body position and removal of tense gas or fluid collections from the pleural space or abdomen.

For most ICU providers, deliberate reduction of FRC by external compression of the rib cage or abdomen is an unfamiliar and somewhat counterintuitive practice. In specific clinical settings, understanding the physiological principles just reviewed should help the clinician to apply and modulate external loads of the chest wall compartments while monitoring central airway pressure with the intent of improving diagnosis and ventilatory management.

### Body positioning

Body positioning, while not commonly perceived as a form of chest wall modification does, in fact, do so, and thereby acts as a powerful complement to regulation of airway pressure for modifying lung mechanics. The fundamentals of prone positioning and its value for ARDS management are well-established [33]. Elevating the head of the bed toward vertical by 15–45°, a standard clinical practice, also modifies the chest wall, partially relieving the lung-compressive effects imposed by the mediastinum, heart, and abdomen. In effect, such positioning ‘unloads’ the lung’s enclosure, predictably increasing trans-alveolar pressures and resting lung volume [48]. Consequently, a more vertical torso inclination tends to simultaneously recruit collapsed lung units while further distending those that are already open. In healthy individuals this generally increases *C*_RC_, *C*_CW_, and *C*_RS_ [18].

Although more upright positioning often improves these same compliance components in disease, such positioning may result in *decreased* lung and respiratory system compliances (*C*_L_ and *C*_RS_, respectively) in a specific subset of patients with severely reduced aerating capacity, including those with severe and unresolving ARDS [49–51]. This unexpected, or ‘paradoxical’, response tends to occur when the capacity to recruit has been exhausted by extreme severity or by a lengthy and unimproving hospital course that allows inflammation to progress from the initial stage of edema and atelectasis to consolidation, fibrosis, and massive loss of functional lung units [9]. In this setting, increased trans-alveolar pressures associated with the more upright position serve only to further distend open lung units without recruitment of additional lung units, shifting the ‘baby lung’ of ARDS into the non-compliant upper range of its pressure–volume curve.

**Fig. 2:**
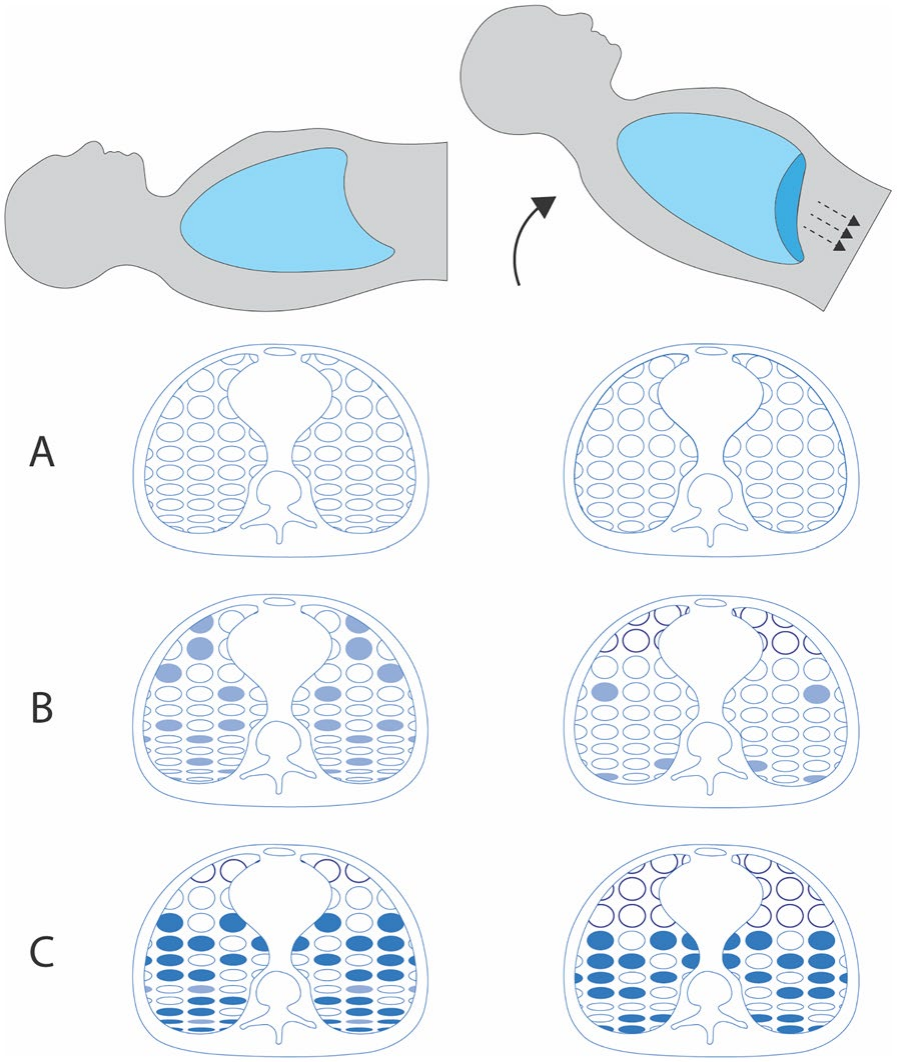
Effect of upright positioning on end-tidal alveolar distension under passive conditions. The weight of the abdomen is off-loaded with upright positioning, resulting in diaphragmatic descent (dashed arrows), increased transpulmonary pressure (*P*_L_), and increased lung volumes. In the ‘healthy’ lung (**A**), chest wall compliance (*C*_CW_) improves, and lung compliance is minimally affected, resulting in improved compliance of the respiratory system (*C*_RS_). In early ARDS (**B**), increased *P*_L_ associated with more upright positioning leads to recruitment of lung units that were previously atelectatic (compressed lung units at the bases) or fluid filled (ovals with light shading). While some end-tidal overdistension may occur in non-dependent regions (ovals with thick outline), recruitment exceeds overdistension, resulting in increased *C*_L_ and *C*_RS_. In late-stage, unresolving ARDS (**C**), there is extensive loss of aeratable lung units as edema and atelectasis are replaced by fibrosis and consolidation (ovals with dark shading). Increased PL in more upright positioning then results in minimal recruitment and widespread overdistension; the improved CCW associated with upright positioning is offset by a relatively greater decline in CL, leading to a paradoxical decrease in CRS

Assuming that the abdomen itself is not constricted in the process, any decrease in *C*_RS_ following placement into a more upright position only occurs if a simultaneous positional decrease in *C*_L_ outweighs the predictable increase in *C*_CW_ that arises from less cephalad diaphragmatic pressure (Fig. 2)*.* In other words, when holding unchanged the *V*_T_ and total PEEP (applied plus auto PEEP), elevations of plateau and driving pressure that occur with upright repositioning are the result of increased end-tidal overdistension prevailing over recruitment. In such patients, more horizontal (flatter) torso positioning will result in decreased plateau and driving pressures as end-tidal overdistension becomes alleviated [51].

### Chest and abdominal weighting

**Fig. 3:**
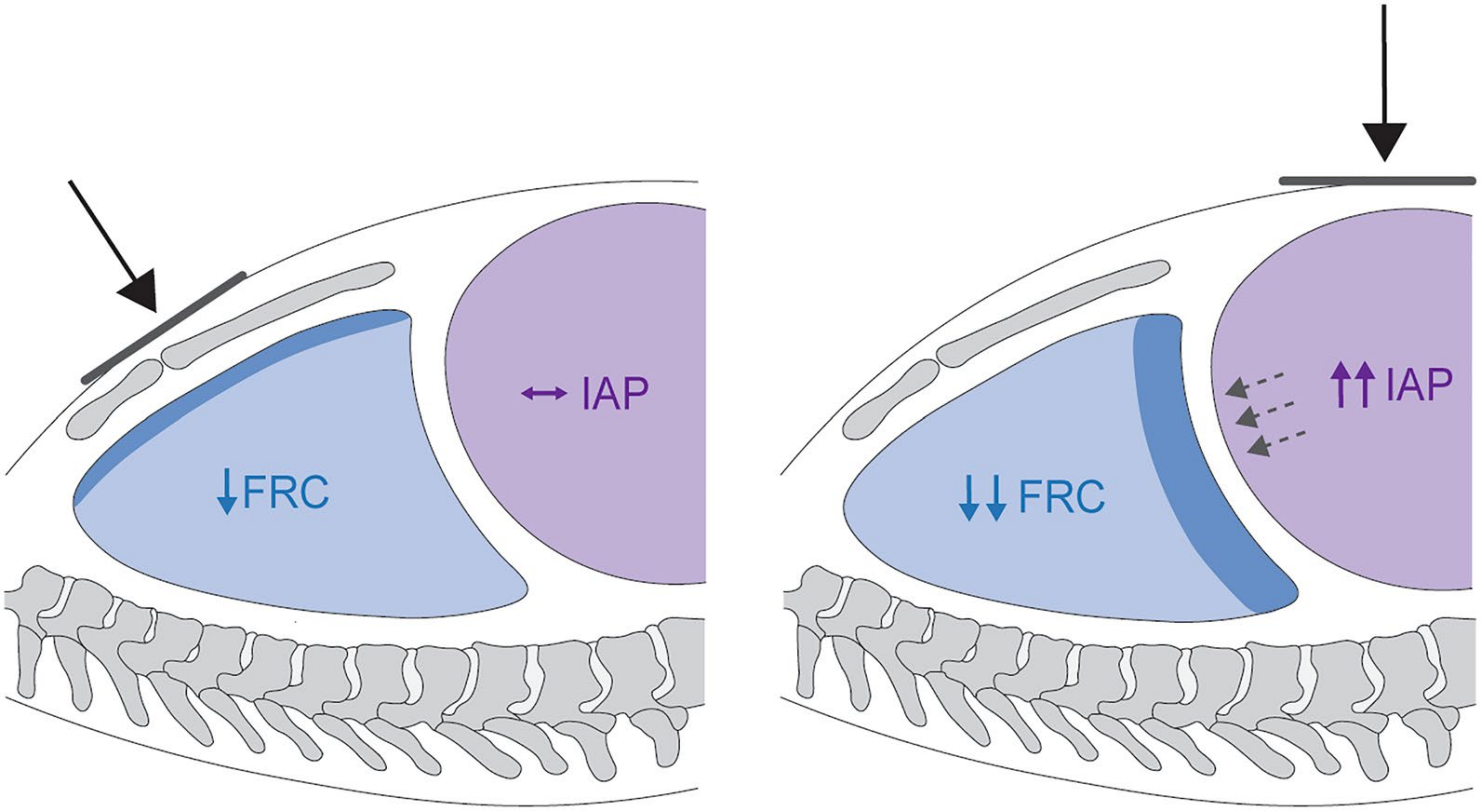
Differential effects of regional chest wall loading. Selective compression of the supradiaphragmatic compartment (left panel) is expected to result in relatively minimal loss (darker blue) of resting lung volume (functional residual capacity, FRC) or change in intra-abdominal pressure (IAP). In contrast, selective compression of the infradiaphragmatic compartment (right panel) increases IAP and displaces the diaphragm cephalad (dashed arrows), resulting in comparatively greater loss (darker blue) of resting lung volume (lighter blue). In the setting of widespread end-tidal hyperinflation with minimal recruitable lung tissue, forced lung volume reduction through either method would be expected to elicit mechanical paradox

Dominance of end-tidal overdistension may also be revealed by maneuvers that impose external pressure on the chest wall, applied upright or supine. The thorax can be compressed along the ventral-dorsal (sagittal) plane by weights that externally load the chest wall to displace its pressure–volume relationship rightward (without necessarily resulting in perceptible change of *C*_CW_, i.e., the *slope* of its tidal pressure–volume curve). Described methods of weighting include placing saline or sand bags over the sternum or abdomen, usually at supine angles approximating 0° to horizontal [7, 8, 10, 11]. These weighting methods apply a vertically directed downward force that partially transmits to underlying lung over a known surface area (‘footprint’) but uncertain internal distribution; nondependent zones closest to the weighted surface are likely to experience a greater degree of compression than dependent zones separated from the external load by greater distance (Fig. 3).

Unlike manual compression, weights are not easily applied when the torso is angulated non-horizontally, and the compressive effect of such weighting varies with the gravitational vector. A pressure-regulated vest, a circumferential rib cage compression device, is not subject to those constraints [52]. In concept, weights may be applied for brief periods for diagnostic purposes, but more commonly in practice have been applied over the anterior rib cage for extended periods with the intent to replicate the benefit conferred by ventral stiffening of the chest wall during prone positioning [53]. In contrast to proning, neither selective loading of the abdomen [54] nor simultaneous weighting of both the chest and abdomen, have been extensively investigated.

### Manual compression

Manual compression over the abdomen or sternum that is sustained through both phases of several tidal cycles is a brief, noninvasive, diagnostic maneuver to detect net end-tidal overdistension [15]. In tracking the behavior of compliance-related measures, such as plateau and driving pressure during passive, volume-controlled ventilation, these compressive maneuvers are virtually always available to the bedside clinician. Acute surgical disruptions of either rib cage or abdominal surfaces, however, might contradict doing so.

Manual compression over either sternum or abdomen alters the chest wall by reducing its compliance throughout the entirety of the tidal breath and/or provides a limiting constraint only to end-tidal distension. As indicated by the foregoing discussion of intercompartmental transmission of pressures, for similar manual efforts an unfluctuating compression of the upper and mid-abdomen, or ‘belly push,’ would be expected to result in greater reduction of the expiratory reserve and resting lung volume (and perhaps less reduction of venous return) than selective compression applied over the rib cage. Therefore, discounting the local effects of a ‘sternal push’, which might favor decompression of disproportionately overdistended ventral lung units [8], the ‘belly push’ may be the preferred technique for eliciting the mechanical ‘paradox’ of improved C_RS_ during a compressive maneuver. It is worth noting that external pushes imposed over the upper or lower back in the prone position may have similar directional effects on *C*_RS_ as do manual compressions in the supine horizontal posture [55]. Independent of site, the external force must reduce end-inspiratory lung volume to observe mechanical paradox.

Precisely how best to conduct these compressive maneuvers is an unsettled question being pursued in ongoing research [15]. Yet, whatever the ventilation mode, *V*_T_, and degree of lung inflation, loading sufficient to reduce lung volume during tidal ventilation can be easily confirmed by demonstrating a significant rise in the plateau airway pressure *during an end-inspiratory breath hold*. With total lung and chest wall volumes unchanged, an upward deflection of the plateau pressure during an end-inspiratory breath hold of ≥ 2 cmH_2_O caused by compressive loading that reverts immediately to baseline following compressive release, indicates the potential of that loading force to reduce end-tidal chest volume when tidal breathing resumes [15, 51, 56], while this is a somewhat arbitrary threshold, it reliably excludes the upward oscillations that may be present during any end-inspiratory breath hold. Similarly, a substantial increase of expired *V*_T_ with the first breath following a loading maneuver (and substantial reduction of expired *V*_T_ with the first unloaded breath) during tidal-volume controlled ventilation also confirms the impact of the compression.

## Clinical considerations

All methods of chest wall loading share the unifying characteristic of altering *P*_L_ and resting lung volume. While their underlying physiologic mechanisms are relatively well-understood, they have not been subjected to rigorous clinical studies that confirm or refute their diagnostic value for guiding safer ventilator adjustments or worth as sustained therapeutic interventions. Nonetheless, solid data regarding compressive mechanics are available that allow prediction of the impact of *P*_L_ alterations on common problem types encountered in intensive care practice. Patients with ARDS, in particular, are proposed as potential candidates for chest wall modification.

In ARDS, tidal closure and re-opening of vulnerable lung units has been identified as a mechanical stimulus for VILI [57]. Indeed, the thrust of most lung protective strategies has been to raise end-expiratory lung volumes to recruit and stabilize collapsible lung units while avoiding excessive driving pressures [1]. In this setting, avoiding hyperinflation of already open lung units is a related but secondary concern. Any intervention that reduces *P*_L_ may encourage collapse of unstable units in ‘recruitable’ lungs; indeed, when unstable lung units are prevalent, encouraging their collapse by horizontal positioning or chest wall restriction is illogical and contraindicated. Illustrating this point, success in simulating the gas exchanging benefits of prone positioning by sustained ventral chest compression has generally been limited or elusive during the early stages of acute lung injury.

In a different micromechanical environment, however (i.e., when unstable lung units do not prevail), the ‘recruitment first’ priority—and, therefore, the treatment perspective—may change. As injury progresses and worsens over time, lung units become less recruitable, the viable baby lung shrinks in capacity, and the tendency for end-tidal hyperinflation poses greater hazard [58]. Improving *C*_L_ by reducing *V*_T_ and/or PEEP simultaneously attenuates barotrauma risk, ventilating stress, strain, and power [59]. In fact, regional end-inspiratory hyperinflation is detectable in severe ARDS even when low *V*_T_ strategies are employed [60]. When sufficiently extensive, limiting such end-tidal hyperinflation by assuming a horizontal position or by external chest compression may not only paradoxically improve tidal *C*_L_ (and *C*_RS_) but also redistribute the *V*_T_ and intrapulmonary perfusion. What benefit—if any—accrues to gas exchanging efficiency by relief of end-tidal hyperinflation remains to be defined.

More horizontal positioning, placement of sternal weights, and active compression of the upper abdomen have been shown to elicit mechanical paradox suggestive of end-tidal overdistension in patients with late-stage ARDS despite the use of *V*_T_ and PEEP presumed to be ‘lung protective’ without it [7, 8, 51, 56]. Potentially, such diagnostic information may help guide wiser selection of ventilator settings, but the therapeutic potential (or consequences) of sustained chest wall modification on gas exchange or clinical outcomes has yet to be established.

## Questions and opportunities for research

Reductions of PEEP and/or *V*_T_ affect the entire respiratory system—both thoracic and abdominal compartments in unison. In contrast, all described methods of chest wall modification alter properties of one compartment disproportionately. Although informative reports have detailed specific clinical responses and physiologic observations in selected populations, numerous questions regarding chest wall modification—many with immediate clinical relevance—remain little researched. For example, although best described in severe ARDS (primarily due to COVID-19), the true prevalence of paradoxical responses to chest wall modification among a general population of patients, including those with and without pre-existing chest wall or lung pathology of any type and severity, is unknown. Randomized trials of chest wall modification that test the clinical value and consequences of detection and reversal of end-tidal overdistension are currently lacking.

Whether for diagnostic or therapeutic purposes, effective chest wall modification depends on reducing end-inspiratory *P*_L_ in zones of end-tidal hyperinflation, an objective that for an unchanging *V*_T_ may be met by reducing FRC or by regional stiffening the chest wall. For exploratory diagnostic maneuvers of brief duration, safety concerns regarding chest wall modifications are few. However, when sustained, such interventions with therapeutic intent have the potential to influence lung architecture, distributions of perfusion and ventilation, hemodynamics, and gas exchange.

The relative efficiency of these compression methods, their optimal execution (‘standardization’), as well as safety, patient tolerance, and durations of any resulting benefit need to be determined. Even fundamental physiologic questions remain only partially answered. For example, how does each method of chest wall modification (sternal weighting, belly push) affect global and regional lung mechanics, hemodynamics, gas exchanging efficacy with variations of PEEP, *V*_T_, and body positioning? Even within the same diaphragm-delineated compartment, external compression is applied over a confined surface footprint; just how widely shared that localized volume restraint may be among contiguous and non-contiguous lung sectors is unknown.

## Summary

Because the lung is surrounded by a regionally diverse and multi-compartmental chest wall, reliance on the plateau and driving pressures that accurately characterize the passive respiratory system may not always reflect the internal environment of the lung. Important physiologic and clinical outcome investigations wait to be conducted to standardize, compare, and prove the value (or non-value) of various chest wall modifications for decision-making and intervention in conditions such as ARDS. Nonetheless, it seems reasonable to propose that detection of unanticipated lung mechanics by a loading maneuver may usefully alert the clinician to reconsider ongoing therapeutic choices for PEEP, *V*_T_, or body positioning.

## Data Availability

Not applicable.

## Abbreviations

ARDS: Acute respiratory distress syndrome
*C*_AB_: Compliance of the abdomen
*C*_CW_: Compliance of the chest wall
*C*_L_: Compliance of the lung
*C*_RC_: Compliance of the rib cage
*C*_RS_: Compliance of the integrated respiratory system
ERV: Expiratory reserve volume
FRC: Functional residual capacity
IAP: Intra-abdominal pressure
*P*_L_: Transpulmonary pressure
*P*_pl_: Pleural pressure
PEEP: Positive end-expiratory pressure
*V*_T_: Tidal volume
